# Reflective Fiber Temperature Probe Based on Localized Surface Plasmon Resonance towards Low-Cost and Wireless Interrogation

**DOI:** 10.3390/s23084165

**Published:** 2023-04-21

**Authors:** Yang-Duan Su, Carter Neal Leatherman, Yuankang Wang, Paul Richard Ohodnicki

**Affiliations:** 1 Mechanical Engineering and Materials Science, University of Pittsburgh, Pittsburgh, PA 15260, USA; 2 Electrical and Computer Engineering, University of Pittsburgh, Pittsburgh, PA 15260, USA

**Keywords:** fiber optic temperature sensor, localized surface plasmon resonance, optical waveguide modeling, reflection fiber probe, transimpedance amplifier, wireless interrogator

## Abstract

Reflection fiber temperature sensors functionalized with plasmonic nanocomposite material using intensity-based modulation are demonstrated for the first time. Characteristic temperature optical response of the reflective fiber sensor is experimentally tested using Au-incorporated nanocomposite thin films deposited on the fiber tip, and theoretically validated using a thin-film-optic-based optical waveguide model. By optimizing the Au concentration in a dielectric matrix, Au nanoparticles (NP) exhibit a localized surface plasmon resonance (LSPR) absorption band in a visible wavelength that shows a temperature sensitivity ~0.025%/°C as a result of electron–electron and electron–phonon scattering of Au NP and the surrounding matrix. Detailed optical material properties of the on-fiber sensor film are characterized using scanning electron microscopy (SEM) and focused-ion beam (FIB)-assisted transmission electron microscopy (TEM). Airy’s expression of transmission and reflection using complex optical constants of layered media is used to model the reflective optical waveguide. A low-cost wireless interrogator based on a photodiode transimpedance-amplifier (TIA) circuit with a low-pass filter is designed to integrate with the sensor. The converted analog voltage is wirelessly transmitted via 2.4 GHz Serial Peripheral Interface (SPI) protocols. Feasibility is demonstrated for portable, remotely interrogated next-generation fiber optic temperature sensors with future capability for monitoring additional parameters of interest.

## 1. Introduction

Embedded temperature sensors that are low-cost and miniaturized to be widely accessible in electrical transmission, energy storage, and power generation facilities have become important to maintain the healthy condition of the device and predict early onset of thermal failures without compromising the general operation and cost of the system. Fiber optic sensors are favored over traditional thermal-resistive-based temperature sensors owing to their compactness by nature and immunity to electromagnetic interference (EMI) in the sensing conditions presented by electric power equipment. In particular, functional thin-film-coated evanescent wave optical fiber sensors receive increasing attention due to their lower fabrication cost compared to fiber Bragg Grating (FBG) or photonic crystal fiber (PCF) temperature sensors, which require expensive femtosecond laser or stacking/drawing equipment to produce the fiber, and their tunability of sensor performance by controlling the doping level and microstructure of the film. Common dielectric materials such as TiO_2_ were studied as functional thin film sensor elements on optical fibers based on the thermal-optic effect of their refractive indices in response to temperature [1]. In the past decade, the mechanism modulated by wavelength shifts due to the change in refractive indices was further explored with the addition of noble metal film layers or metal NP to various dielectric material hosts to induce surface plasmon resonance (SPR) or LSPR, respectively, creating resonance peaks that can be used as the primary interrogable spectral feature [2,3,4,5,6,7,8,9].

LSPR responses have been demonstrated to be tunable in terms of spectral positions and intensity by modifying precious metal doping levels and heat-treating temperature to control particle size [10,11,12,13], and by changing the refractive index of the dielectric host, such as using oblique deposition methods for the sensing films [14]. LSPR is a particular type of SPR that happens when the propagating distance of surface plasmon polaritons is confined to a scale comparable to the wavelength of incident light [15]. Unlike SPR, it does not require total internal reflectance of propagating light to occur, making LSPR less stringent to be produced and more readily leveraged in engineering of optical materials. Not only were Au-incorporated nanocomposite films systematically characterized to assess the potential sensing performances [16,17], but evanescent wave fiber optic temperature sensors were also demonstrated to adopt Au NP in an oxide matrix as a cladding to detect temperature variations based on shifts in optical transmission spectra [18,19,20,21]. However, most works on plasmonic fiber optic sensors have focusing on using the mechanism of SPR wavelength shifts, which is a result of refractive index change [22,23,24]. Interrogation with wavelength-shifts associated with modifications to a real refractive index may suffer from cross-sensitivity issues due to the fact that it is sensitive to any measurands which impact the real index [25]. In addition, the cost of spectral shift-based interrogators is substantially greater than that of intensity-based interrogation systems due to the need for low linewidth sources and high wavelength resolution detection circuits. In contrast, LSPR intensity modulation has the potential to discriminate multiple parameters with relatively low cost and simple optical interrogation systems, because of the local change in spectral bands or peaks in respond to each measurand. Nevertheless, a potential drawback of intensity-based sensors can be the fact that their accuracy is highly dependent on the stability of the light source, which can be mitigated by multi-wavelength referencing or monitoring the light source intensity.

Prior work successfully demonstrated intensity-based Au/SiO2 LSPR optical fiber temperature sensors in the transmission configuration with an intuitive interpretation of sensor responses based upon the modified film LSPR absorption [26]. However, fiber sensors configured as reflection probes offer distinct advantages in installation during in-operando monitoring of energized electric power facilities such as high-voltage transformers. Naci Inci et al. [27] demonstrated one of the first functional material-based reflection fiber temperature probes with a TiO_2_ coating of 1 μm on the tip of a single-mode fiber, where the temperature-dependent optical phase change within the TiO_2_ film is a function of the thermal-optic coefficient and thermal expansion coefficient of the thickness of the film. The results showed decreased reflectance at a single wavelength of 780 nm as the temperature increased. In our work, we demonstrated the first reflection intensity-modulated fiber optic temperature sensor by using a thin film composed of an Au-incorporated nanocomposite, with absorption bands displaying temperature-dependent characteristics of LSPR. The intensity-based temperature dependence is dominated by the resistivity, $\rho_{Au}$, of Au NP, and is further modified by the optical constant of the matrix phase and thermal expansion. As described in (1), by equating the definition of resistivity of metal to its correlation with drift mobility of free electrons [28], the increased electron–electron and electron–phonon scattering at the surface and bulk film under elevated temperatures increases $\rho_{Au}$. This results in an increase in electron and phonon collision frequency, $\omega_{c}$, which dampens the intensity of LSPR absorption band and modifies the reflectance of the film. $m_{e}^{*}$ is the effective mass of free electron, *e* is electronic charge, *T* is temperature, *N* is electron density, and *C* is an independent constant.

(1) $$\rho_{Au}=\frac{m_{e}^{*}T}{e^{2}NC}=\frac{m_{e}^{*}\omega_{c}}{e^{2}N}$$

A less obvious temperature effect is the red shift of the LSPR peak with increasing temperature due to the increase in the refractive index of the matrix phase originating from the change in electronic polarization of the dielectric oxide with temperature. From the fiber optic interrogation perspective, interrogators and light sources dominate system cost and prevent fiber sensor probes being integrated into commercial power equipment [29]. Raghavan et al. [30] designed a wavelength-resolving detector that integrates a position-sensitive photodetector with a dispersive-filter-coated detector to serve as a low-cost FBG interrogator. Our previous works proposed a design of low-cost and wireless interrogation based on a photodiode-TIA circuit that converts optical signals into simple electrical signals [31]. Here, we further incorporate a dual-stage amplifier design with a low-pass filter to account for temperature response time and optical response resolution of the plasmonic sensor. This wireless communicated interrogation is intended for field deployment of intensity-based fiber sensors in medium/high voltage electrical assets, where remote monitoring without electrical wires to access is a personnel and equipment safety requirement. Taken together, to emphasize the practical advantage of reflection-based fiber sensors and wireless interrogation, and the cost-effectiveness of intensity-based sensor/interrogator, this work aims to provide a theoretical guide towards reflection-mode optical waveguide modeling and experimental validation. This is examined in the context of AuNP-incorporated oxide LSPR temperature fiber sensors when combined with a wireless signal demodulation circuit, which is an area that currently lacks discussion in the literature for these sensing materials on an optical fiber platform.

## 2. Materials and Experimental Procedures

### 2.1. Materials Preparation and Characterization

**Figure 1:**
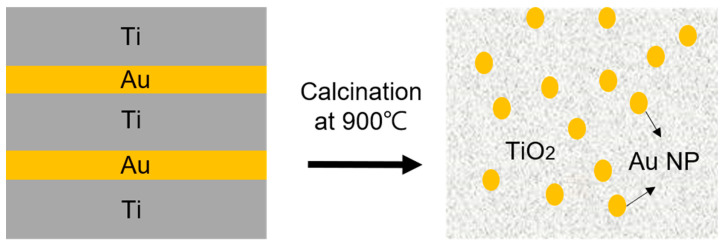
Schematic of example multilayered film stack to achieve uniformly distributed Au NP in the TiO_2_ matrix after calcination.

Au/TiO_2_ and Au/SiO_2_ nanocomposite thin films are made by E-beam evaporation (PLASSYS, Marolles-en-Hurepoix, France) and magnetron RF sputtering (Angstrom, Cambridge, Ontario, Canada), respectively, in periodic multilayered structures. In preparing Au/TiO_2_ films, three layers of titanium are evaporated at a rate of 0.01 nm/s for 15 nm each, with two layers of gold alternately evaporated in between at a rate of 0.05 nm/s for 4.4 nm each. The specified thicknesses are to ensure an approximate 10 vol% of Au after calcination step considering the volume expansion from titanium oxidation. This process is performed in a vacuum with a plasma etch cleaning step preceding the deposition. To reach oxidation and spheroidization of fully dispersed Au NP, the Au/Ti film stacks are then subjected to a post-calcination step in a tube furnace from room temperature to 900 °C in air at a ramping rate of 2.5 °C/min, followed by isothermal hold at 900 °C for 2 h and a cooling step to room temperature at a rate of 2.5 °C/min. Figure 1 illustrates the deposition and post-processing of an example Au-NP-incorporated TiO_2_ thin film. In preparing Au/SiO_2_, three layers of SiO_2_ are sputtered at a rate of 0.012 nm/s for 25 nm each, with two layers of gold alternately sputtered in between at a rate of 0.05 nm/s for 4.5 nm each. The working pressure in the sputtering chamber is set to be 5 × 10^−3^ Torr with an Argon working gas at a flow rate of 10 sccm. The Au/SiO_2_ film stacks are heat-treated after the deposition using the same calcination schedule described above. All nanocomposite thin films are coated on both 1-inch quartz disc and step-index multimode (MM) fiber (FG105UCA, Thorlabs, Newton, NJ, USA) substrates. MM fiber segments 8 cm long are cleaved on one end. Both the fiber and planar quartz substrates are then cleaned with IPA and ethanol before deposition. To deposit films on the fiber tip, during all processes in the above deposition chambers, fiber segments are held upright by Kapton tapes with the cleaved end facing the target materials.

**Figure 2:**
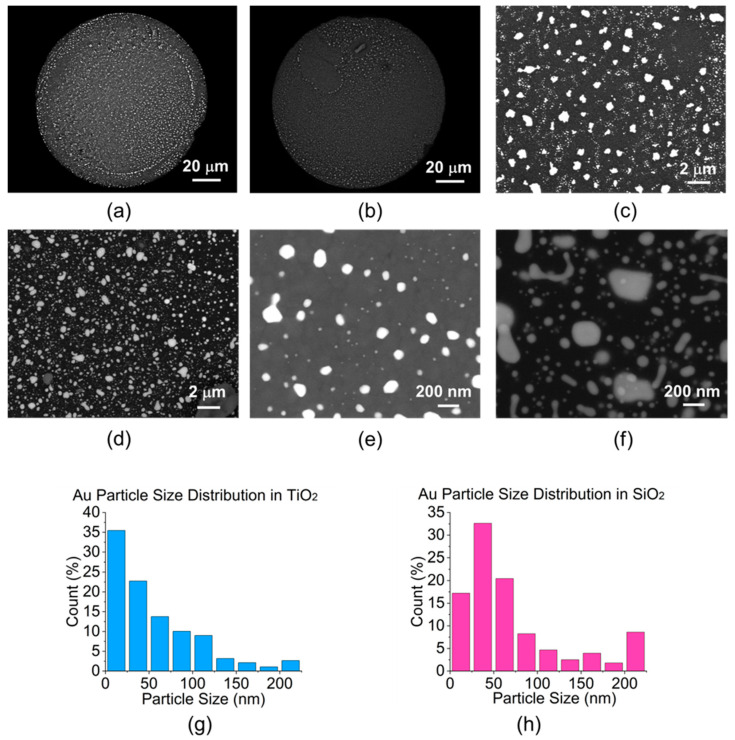
Low-vacuum backscattered-electron SEM images of (**a**) Au/TiO_2_ and (**b**) AuSiO_2_ of a fiber sensor tip. Uniform Au particle distribution at the surface of (**c**) TiO_2_ and (**d**) SiO_2_. Spherical inclusions of Au NP in (**e**) TiO_2_ and (**f**) SiO_2_ at the nanoscale, with SiO_2_ showing more diverse shapes of elongated Au particles. Au NP size distribution in (**g**) TiO_2_ and (**h**) SiO_2_ matrix.

The driving force for Au NP formation in the oxide matrix is a combined effect of solid-state interfacial diffusion and a difference between bond energies of Au-Au and Au-oxide. Figure 2 shows the SEM (ZEISS, Oberkochen, Germany) images of the calcined fiber samples with Au/TiO_2_ or Au/SiO_2_ film deposited on the fiber tip. All SEM images are performed in a variable-pressure mode with a low vacuum level at a chamber working pressure of 4 × 10^−4^ to 7 × 10^−4^ mbar to prevent the charge accumulation on the non-conductive oxide sample surfaces. Au/TiO_2_ and Au/SiO_2_ film on a fiber sensor tip are characterized in Figure 2a and Figure 2b, respectively. Although larger particles of approximately 1 μm can be observed on the surface, Au particles appear to be uniformly dispersed as shown in Figure 2c,d for both materials, with the majority of particles being much smaller in size. Nanoscale Au spherical particles ranging from 100 to 200 nm can be seen in Figure 2e on the surface of the Au/TiO_2_, whereas in Figure 2f, more elongated and islanded shapes of Au particles are revealed on the surface of the SiO_2_ host. This is likely due to the absence of grain boundaries in the amorphous SiO_2_ to effectively trap the NP on the surface of the films. However, Au particle size distribution in Figure 2g,h shows that much smaller (<50 nm) Au NP dominate in both the TiO_2_ and SiO_2_ matrix hosts, which accounts for 57.5% of the NP in TiO_2_ and 50% of that in SiO_2_ matrices. This meets the quasi-static approximation of propagating light wave in nanostructures and confines the LSPR absorption bands in the visible range.

**Figure 3:**
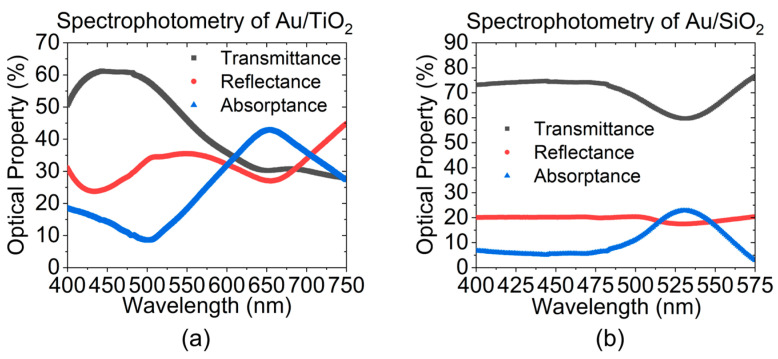
Spectrophotometry of (**a**) Au/TiO_2_ and (**b**) Au/SiO_2_ nanocomposites on planar quartz substrates showing representative LSPR absorption bands in the visible range.

The larger particles tend to lie on the surface, resulting from enhanced surface diffusion rates of Au atoms relative to the film interior. The optical properties of both materials are first confirmed using planar quartz samples measured by standard spectrophotometer (JASCO, Japan). As shown in Figure 3a, the Au/TiO_2_ sample exhibits an LSPR absorption band with full width at half maximum (FWHM) spanning from 600 to 750 nm. Spectra of Au/SiO_2_ in Figure 3b, on the other hand, have the FWHM of LSPR band at 500 to 550 nm. Despite the fact that most Au NP are spherical and less than 50nm in diameter, the less symmetric and larger ones will tend to contribute additional absorption and even scattering at longer wavelengths than would be predicted within the quasi-static approximation, thereby shifting the spectral location of the LSPR and affecting the intensity of the absorption peak [32]. This tends to raise heterogeneities among samples with the same processing recipe. In addition to particle size, different spacing, resulting from plasmon coupling and radiative dipole interaction between nearby Au NPs, is another parameter to consider that can vary the spectral location of LSPR peak [33]. A systematic analysis is required regarding the size, shape, and spacing distribution of the NP inclusions and their effect on the effective optical constants of the nanocomposite medium, and thus the LSPR spectral properties, for high quantitative accuracy of LSPR optical response modeling. Nevertheless, the quasi-static approximation can still yield useful insights for comparative benchmarking with experimental results and trends observed in practice.

### 2.2. Sensor and Conventional Interrogation Setup

**Figure 4:**
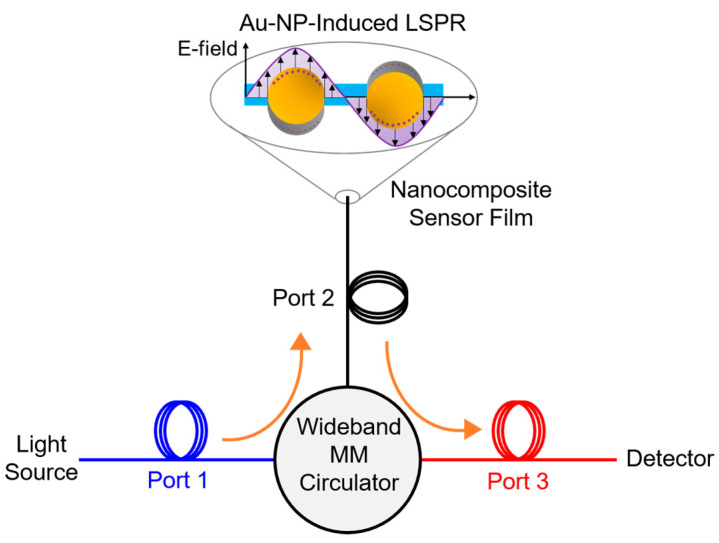
Optical circulator configuration of reflection-based sensor probe with fiber tip functionalized by Au-incorporated nanocomposite that shows LSPR absorption features. Conceptually, the purple arrows indicate the coherent oscillation of free electrons in Au NPs with charge separation, while the blue surface represents the oxide matrix surrounding the Au NPs.

**Figure 5:**
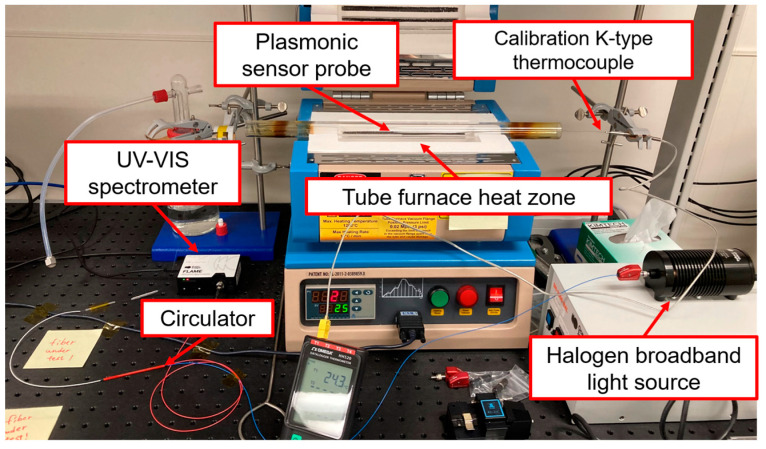
Experimental setup of plasmonic sensor probe temperature sensing.

The reflection-based fiber sensor is made by fusion-splicing a MM fiber sensor segment, 105 μm in core diameter, to a wideband MM circulator (WMC1H1S, Thorlabs, Newton, NJ, USA), as shown in Figure 4. Lead fibers at port 1, 50 μm in core diameter, are connected in free space to the light source; whereas fibers at port 2 and 3, with core diameter of 105 μm, are spliced and connected to the sensor element and the interrogator, respectively. Temperature sensing experiments are performed using the conventional interrogation equipment: a Halogen broadband white light source (Ocean Insight, Orlando, FL, USA) with Flame UV-VIS spectrometer (Ocean Insight, Orlando, FL, USA). As shown in Figure 5, the optical fiber is inserted in a tube furnace (MTI, Richmond, CA, USA), with the sensor probe being placed at the center of the heating zone. A k-type thermocouple is placed from the other end into the quartz tube in vicinity of the fiber sensor to calibrate the temperature readings. The furnace is programmed to ramp from room temperature to 500 °C in 30 min, with an isothermal hold for 10 min and subsequently cooled down by air. The data sampling rate of the thermocouple logger is set to be 1 s^−1^. Data acquisition parameters of the spectrometer depend on the reflectance of the post-annealed nanocomposite deposited on the sensor tip. For Au/TiO_2_ sensors, the integration time is set to be 3 to 5 ms. However, 10 to 12 ms of integration time is needed for Au/SiO_2_ sensors due to the lower reflection intensity of the SiO_2_. For all sensors in this setup, 100 scans of spectrums are averaged to obtain one visible range of spectral data. The data update rate is set to be 1 s^−1^. The spectral resolution of the spectrometer is 1.35 nm with a boxcar averaging width of 5 data points being applied to increase the signal-to-noise ratio (SNR).

### 2.3. Wireless Interrogation

**Figure 6:**
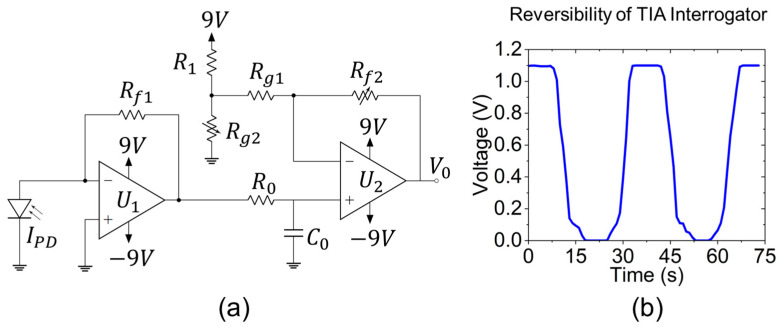
(**a**) Circuit diagram of the dual-stage transimpedance amplifier. (**b**) Reversibility of low-cost interrogator voltage signal tailored to the baseline reflectance of the reflective fiber sensor.

**Figure 7:**
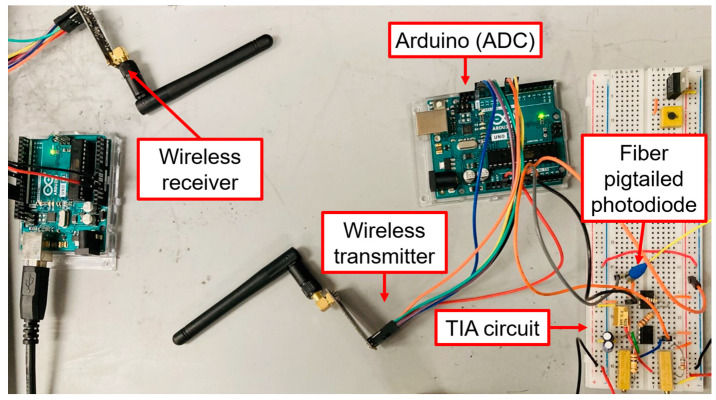
Entire setup of the low-cost and wireless interrogator circuit.

The low-cost interrogator circuit consists of a pigtailed photodiode (FDSP625, Thorlabs), dual-stage amplifiers, two Arduino Uno microcontroller boards, and two wireless transceiver modules (nRF24L01+), with a total build cost of ≤USD 250. The detailed operation principles of TIA was described previously in our work [31]. Here, an updated design of the dual-stage TIA is shown in Figure 6a. A single wavelength fiber-coupled LED (M530F2, Thorlabs) is used as the light source. Briefly, the photocurrent, $I_{PD}$, from the diode is converted into output voltage, $V_{0}$, by the operational amplifier (Op-Amp), and later transmitted by the microcontrollers. The max. and min. of the photocurrent and output voltage define the gain of the Op-Amp, which is also represented by the feedback resistor. The photocurrent change from the sensor is designed to be amplified to the voltage input range of the Arduino analog-to-digital converters (ADC), and a DC offset is added for the minimum current to correspond to the minimum voltage of the ADC. The internal ADC reference voltage of the Arduino is 1.1 V. The dual-stage amplifier consists of a high-gain transimpedance amplifier, $U_{1}$, cascaded into a low-gain amplifier, $U_{2}$, with filtering and a DC offset [34]. The cut-off frequency of the low-pass filter is defined by $R_{0}$ and $C_{0}$ to capture the low frequency of temperature optical response. The DC offset is controlled by resistors $R_{1}$ and $R_{g2}$ that form a voltage divider, and the gain is customized by resistors $R_{f2}$, $R_{g1}$ and $R_{g2}$ through two potentiometers. The non-inverting input of $U_{2}$ takes the voltage produced by the first stage, and its inverting input receives voltage produced by the voltage divider. With the component values chosen, this circuit can provide signal gain between 0.07 V/nA and 0.33 V/nA and up to 90V of negative DC offset. For a proof of concept, the nanocomposite reflection fiber sensor is spliced with the photodiode and integrated with the low-cost customized TIA interrogator to test the voltage output. Figure 6b shows the reversibility of the voltage variations wirelessly transmitted as a function of input optical intensity by manual adjustment of the LED driver over a short period of time. The transmitter-integrated Arduino is programmed to take 100 voltage samples each second and average them to mitigate the noise inherent to a high-gain system. The nRF24L01+ wireless modules communicate with the Arduino through an SPI interface and transmit/receive signals in the 2.4 GHz band. Data is collected and logged from the receiver using the USB interface of the Arduino at a sampling rate of 1 s^−1^ after the data pre-processing. The entire low-cost wireless interrogator is presented in Figure 7. Two 9 V batteries are connected in series to power the entire interrogator circuit. Alternative power supply options are being considered for future prototypes.

## 3. Theoretical Modeling Procedures

In order to benchmark and validate the experimental results of LSPR plasmonic sensors, we developed an analytical model that combines thin-film optics and an optical waveguide in reflection geometry carried out in MATLAB programming. This model consists of two major parts: optical constant models of different Au-incorporated nanocomposites in a visible range and an MM fiber wave optics model that starts from a collimated light source through the lead fiber to the nanocomposite film, implemented by Fresnel and Airy’s expression of transmission and reflection coefficients in layered media.

### 3.1. Optical Constant Modeling

**Figure 8:**
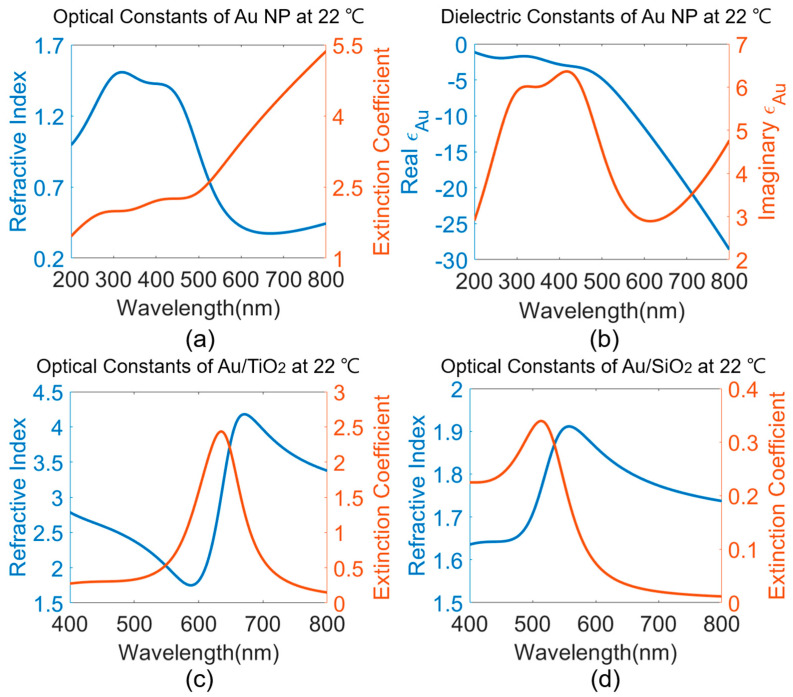
(**a**) Optical constant model, (**b**) associated real and imaginary dielectric constants of Au nanoparticles at room temperature. Effective optical constant model of (**c**) Au/TiO_2_ nanocomposite, and (**d**) Au/SiO_2_ nanocomposite simulated at room temperature.

Optical constant models using Sellmeier dispersion relations of SiO_2_ and TiO_2_ were described in detailed in our previous work [31], with the degree of birefringence of TiO_2_ polycrystalline tetrahedral structure neglected. Constant thermo-optic coefficients of 1.28 × 10^−5^/K and −1.49 × 10^−4^/K are used for SiO_2_ and TiO_2_, respectively, to capture the temperature dependence of refractive indices [20,27]. On the other hand, optical constants of Au NP, $n_{Au}$, requires a careful consideration of both the electronic intraband absorption in the longer visible range and the interband absorption in the shorter, visible-to-UV range. This is modeled by a linear combination of interband transition term of the dielectric constant of Au, $\varepsilon_{Au}^{IB}$, and a Drude oscillator in (2). $\varepsilon_{\infty }$ is the high frequency limit of dielectric constant of Au. The asymmetric line shapes of the two interband transition peaks render it difficult to model $\varepsilon_{Au}^{IB}$ without using many Lorentz oscillators with phenomenological fitting parameters. Therefore, critical point analysis of interband transitions [35] are performed in (3) with parameters acquired from Etchegoin et al. [36], where $C_{j}$ is the amplitude, $\varphi_{j}$ the phase, $E_{j}$ the interband energy gap, $\tau_{j}$ the broadening parameter for the *jth* critical point, and $\mu_{j}$ the order of the pole. As simulated in Figure 8a, two dominant interband electronic transition features are captured in the 300 to 400 nm region. The same interband transition features can be seen in the corresponding complex dielectric function of Au NP in Figure 8b. The temperature dependence of Au NP lies in the Drude term in (2), where $\omega_{p}$ is plasma frequency as a function of temperature and a thermal expansion coefficient of Au, $\gamma_{e}$, as in (4) [37]. ω is the frequency components of incident light, and $\omega_{c}$, in (5), is a size-dependent collision (damping) frequency as a result of the electro–electron scattering frequency, $\omega_{ce}$, and the electron–phonon scattering frequency, $\omega_{cp}$. $v_{F}$ is the Fermi velocity representing free electrons travelling in Au, and $r_{np}$ is the Au nanoparticle size. Electro–electron scattering in (6) is modeled by the Lawrence expression [38], whereas the Holstein model in (7) is based on the Debye model of phonon’s specific heat contribution in solids and is used to model electron–phonon scattering [39]. Γ is scattering probability averaged over the Fermi surface, ∆ is Umklapp fractional scattering, $E_{F}$ is the Fermi energy, *h* is the Dirac constant, $k_{B}$ is the Boltzmann’s constant, $T_{D}$ is the Debye temperature, and $\omega^{0}$ is a frequency constant.

(2) $$n_{Au}\left(\omega ,T\right)=\sqrt{\varepsilon_{Au}^{IB}+\varepsilon_{\infty }-\frac{\omega_{p}^{2}}{\omega \left(\omega +i\omega_{c}\right)}}$$

(3) $$\varepsilon_{Au}^{IB}=\sum_{j=1}^{2}C_{j}\left[e^{i\varphi_{j}}{\left(E_{j}-E-i\tau_{j}\right)}^{\mu_{j}}+e^{-i\varphi_{j}}{\left(E_{j}+E+i\tau_{j}\right)}^{\mu_{j}}\right]$$

(4) $$\omega_{p}\left(T\right)=\omega_{p}^{o}{\left[1+\gamma_{e}\left(T-298\right)\right]}^{-1/2}$$

(5) $$\omega_{c}\left(T\right)=\omega_{ce}\left(T\right)+\omega_{cp}\left(T\right)+\frac{v_{F}}{r_{np}}$$

(6) $$\omega_{ce}\left(T\right)=\frac{1}{6}\pi^{4}\frac{\Gamma ∆}{h\;E_{F}}\left[{\left(k_{B}T\right)}^{2}+{\left(\frac{h\omega }{4\pi^{2}}\right)}^{2}\right]$$

(7) ωcp(T)=ω0[25+4(TTD)5 ∫0TDTz4 dzez−1]

(8) $$\frac{\varepsilon_{eff}-\varepsilon_{oxide}}{\varepsilon_{eff}+2\varepsilon_{oxide}}=f_{Au}\frac{\varepsilon_{Au}-\varepsilon_{oxide}}{\varepsilon_{Au}+2\varepsilon_{oxide}}$$

To derive the optical constant models for Au–oxide nanocomposites, Maxwell–Garnet effective medium theory, (8), is used. $\varepsilon_{eff}$ gives the effective complex dielectric constant of the nanocomposite with Au NP assumed as spherical inclusions for simplicity, where the depolarization factor equals to 1. $f_{Au}$ is the volume fraction of Au NP doped in the oxide matrix. A total of 10 vol% of Au NP is assumed for all simulations in this work due to the temperature sensitivity analysis in our previous work [31] and the mechanical strength of the actual nanocomposite film studied by Rodrigues et al. [40]. Using Equation (8) and the parameters described in Table 1, the resulting optical constants of Au/TiO_2_ and Au/SiO_2_ are shown in Figure 8c,d. At the same Au doping level, Au/TiO_2_ shows a larger extinction coefficient in the anomalous dispersion region than Au/SiO_2_ due to the larger real index of TiO_2_ incorporated in the Maxwell–Garnet equation and the lack of an interband electron absorption overlaying the LSPR.

### 3.2. Optical Waveguide Modeling

**Figure 9:**
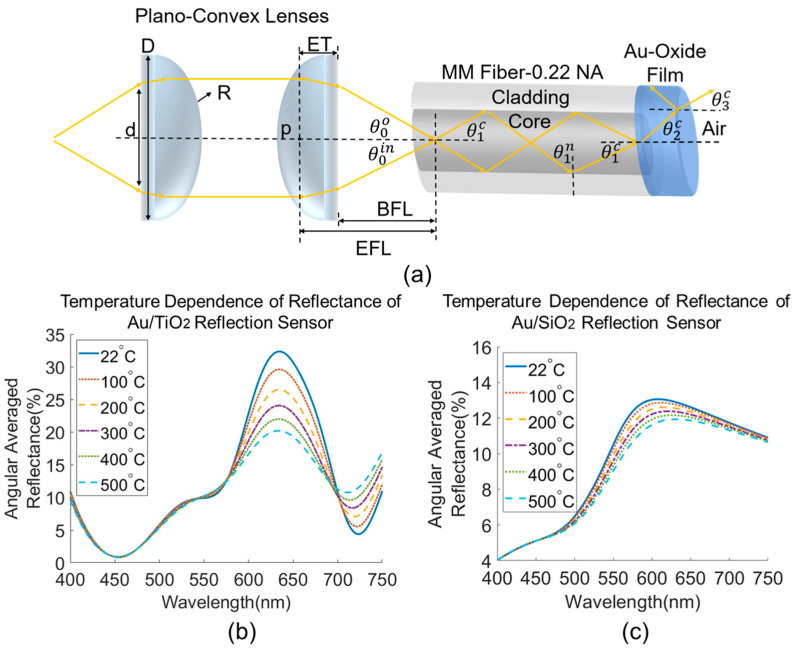
(**a**) Graphical illustration of optical waveguide model of Au/Oxide nanocomposite-coated sensor in reflection geometry, assuming free space coupling of plano-convex lenses. Simulated temperature dependence of the absolute reflectance averaged at a range of incident angles for (**b**) Au/TiO_2_ sensor and (**c**) Au/SiO_2_ sensor.

Figure 9a illustrates the optical waveguide model of a fiber optic reflection sensor. The first step of the model assumes a broadband light source that launches through a pair of plano-convex lenses. These lenses are assumed to be made of MgF2-coated N-BK7 glass with a real index of 1.5 and a diameter, *D*, of 12.7 mm. Lenses are assumed to be coupled with the lead fiber in the air. The numerical aperture (NA) of a lens is a function of its refractive index, radius of curvature, *R*, and the emission diameter, *d*, of the light source. The effective focal length (EFL) of the lens is defined by the lensmaker’s equation [41], and the back focal length (BFL) can be found by assuming a constant value for the edge thickness (ET) of the lens. ET is the thickness of the lens to a principal plane, *p*, where the light mode bends due to refraction in the lens. By requiring that the NA of the MM fiber, 0.22, equals to that of the plano-convex lenses, the emission diameter of the source can be determined to form a trigonometric relation with BFL. This derives and confines the incident angle into the fiber to a specific range, which simulates core modes transmitting through the lead fiber of the sensor. A multilayered media can be envisioned when the core modes enter the lead fiber, creating three interfaces: air-core, core-film, and film-air. Each interface is conditionally implemented by Snell’s law and critical angle calculation if total internal reflection can be satisfied. This determines the refraction angle of every opposing medium. For simplicity, we assume the optical loss due to core-cladding mode coupling is negligible and each core mode is propagating independently throughout the process. From light source to detector, the roundtrip propagation of a complete optical signal can be simulated at three steps: forward propagation at the entrance fiber, propagation and multi-reflection within the nanocomposite sensor film, and backward propagation at the exit fiber connected to the detector. The final total reflectance of the fiber sensor received by the detector is estimated as:

(9) $$R_{total}=T_{f}\cdot R_{film}\cdot T_{b}$$

where $T_{f}$ and $T_{b}$ are transmittances calculated at the air/core interfaces at the entrance and exit, respectively. Assuming isotropic and linear wave optics, this is implemented using simple Fresnel transmission and reflection coefficients to derive the corresponding transmittance and reflectance. However, $R_{film}$, the effective reflectance of the silica core/film/air media, entails a careful consideration because of the finite thickness of thin solid films falling into the scale of the coherence length of propagating light wave. This condition is described in (10), where l is the thin film thickness, λ the incident wavelength, $n_{2}$ the complex optical constants of the film, ∆λ the spectral bandwidth of the light source [42].

(10) $$l\leq \frac{\lambda^{2}}{2\pi n_{2}∆\lambda }$$

(11) $$t=\frac{t_{12}t_{23}e^{-i\varphi }}{1+r_{12}r_{23}e^{-2i\varphi }}$$

(12) $$r=\frac{r_{12}+r_{23}e^{-2i\varphi }}{1+r_{12}r_{23}e^{-2i\varphi }}$$

(13) $$\varphi \left(T\right)=\frac{2\pi ln_{2}cos\theta_{2}^{c}}{\lambda }\left[\frac{1}{l}\frac{dl}{dT}+\frac{1}{n_{2}}\frac{dn_{2}}{dT}\right]$$

(14) $$R_{film}={\left|r\right|}^{2}$$

(15) $$T_{film}=\frac{n_{3}cos\theta_{3}^{c}}{n_{1}cos\theta_{1}^{c}}{\left|t\right|}^{2}$$

The multiple internal reflections within the film contribute to both the effective transmission coefficient and reflection coefficient of the thin film in (11) and (12), respectively. They are also called the expressions of Airy’s summation [43], which consist of: $t_{12}$ and $r_{12},\;$ the simple Fresnel transmission and reflection coefficients at the core/film interface; and $t_{23}$ and $r_{23},\;$ those at the film/air interface illustrated in Figure 9a. The resulting phase gain, φ, from the thin film propagation is described in (13) as a function of temperature. This temperature dependence comes from both the thermo-optic effect ($\frac{dn_{2}}{dT}$) of the complex optical constants described in the previous section and the thermal expansion ($\frac{dl}{dT}$) of the thickness of the film [27]. The thermal expansion coefficient used for TiO_2_ is 7.14 × 10^−6^/K [27], and 2.4 × 10^−7^/K for SiO_2_ [44]. The baseline thicknesses of the simulated Au-oxide nanocomposite films are assumed to be 85 nm at room temperature, which are approximately the same as the film fabricated in the experimental recipe and measured in the subsequent sample characterization. S-polarized and p-polarized waves are both considered in deriving the simple Fresnel coefficients, which gives two sets of Airy’s effective transmission and reflection coefficients for later being averaged in (14) to obtain the effective reflectance and in (15) for effective transmittance. The simulated reflection fiber sensor absolute reflectance in response to temperature is shown in Figure 9b,c for Au/TiO_2_ and Au/SiO_2_ nanocomposite functional coatings. The Au/TiO_2_ sensor shows a higher temperature response in the relevant LSPR wavelength region than the Au/SiO_2_ sensor. This is a direct result of the refractive index of the polycrystalline TiO_2_ being 1.5 to 2.5 times the index of amorphous SiO_2_ in the visible range. In theory, the Au/TiO_2_ is thus expected to exhibit a higher temperature sensitivity than AuSiO_2_ in reflectance of a reflection configured fiber optic sensor at the same Au nanoparticle loading.

## 4. Results and Discussion

**Figure 10:**
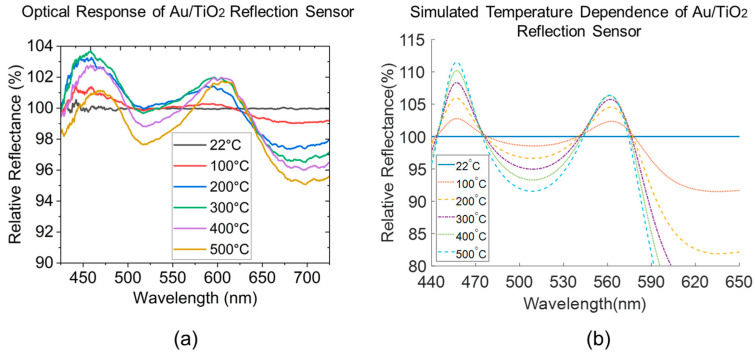
(**a**) Experimental results of the optical response of an Au/TiO_2_ plasmonic reflection sensor under temperature sensing test. (**b**) Simulated optical response to temperature of the same using the optical waveguide model.

**Figure 11:**
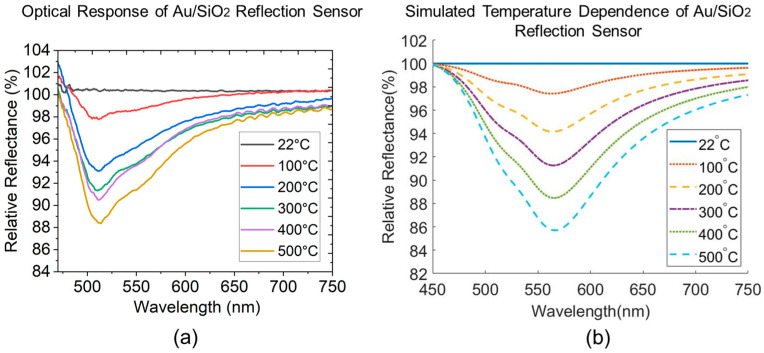
(**a**) Experimental results of the optical response of an Au/SiO_2_ plasmonic reflection sensor under temperature sensing test. (**b**) Simulated optical response to temperature of the same using the optical waveguide model.

The temperature sensing experiments are conducted in real-time using the furnace setup described in Section 2.2. to compare the plasmonic-induced temperature sensitivity of the two reflective nanocomposites and validate against their theoretical performance modeling results. To simulate the spectral baseline of the interrogation software (OceanView 2.0), the comparative modeling results are completed by assuming that 100% of the absolute reflectance of the Au–Oxide reflection sensor modeled at 22 °C is served as the baseline reference. The subsequent temperature-dependent spectra at all elevated temperatures are obtained by dividing the modeled absolute reflectance at those temperatures by the baseline and normalized with a value of 100% to complete the relative reflectance. Figure 10a shows the experimental results of an Au/TiO_2_ reflection sensor changing as the environmental temperature increases from room temperature to 500 °C. All spectral convex features within the visible region are captured and confirmed experimentally when compared to their simulation counterpart in Figure 10b. The characteristics in the visible range are dominated by the LSPR absorption of Au NP inclusion, with the one from 600 nm and onwards being dominating, which can be observed from the absolute reflectance spectrum in Figure 9b. As for the absorption band before 600 nm, the convex-upward band at 500 to 550 nm decreases in intensity with temperature. The convex-downwards feature at 450 to 500 nm and 550 to 600 nm are shown to be increasing with temperature in the modeling but is observed to first increase then drop in relative reflectance at higher temperatures due to the spectral damping effect from the 500 to 550 nm band being in the immediate vicinity. Although the experimental results validate the spectral feature locations for the Au/TiO_2_ sensor, the peak change in reflection intensity is lower than the model in theory would predict. We hypothesize that these intensity deviations derive from the discrepancies between the assumed uniform film thickness, neglected TiO_2_/air surface and TiO_2_/fiber interface roughness, average particle size and spherical shape of Au NPs in the model and those formed in the fabricated sample, as well as the polycrystalline microstructure of the Au/TiO_2_ sample. However, the Au/SiO_2_ shows good agreement both in terms of the intensity change and the major LSPR peak, as shown in Figure 11a,b. At low temperatures, 22 to 200 °C, the Au/SiO_2_ shows a higher approximately linear response with a temperature sensitivity of 0.04%/°C, which is the temperature range of interest for electric power equipment thermal health monitoring. The convex-upward band at 500 to 600 nm has an accuracy of relative reflectance intensity change within ±3% when being benchmarked against the simulated intensity change in Figure 11b. Both sensing layers in the reflection fiber optic configuration show potential for temperature sensing in normal operational temperature rises in electrical power equipment, as well as high temperature monitoring in applications under extreme conditions. This could be relevant for a wide range of other harsh environment applications including aviation aerospace, power generation, and industrial manufacturing processes.

**Figure 12:**
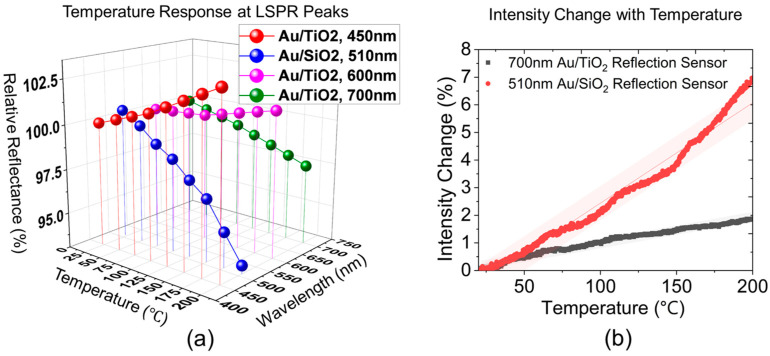
(**a**) Temperature responses demodulated at individual LSPR peaks of Au/TiO_2_ sensor compared to the dominant LSPR peak (510 nm) of Au/SiO_2_ sensor. (**b**) Percent change in relative reflectance of Au/TiO_2_ at 700 nm compared to Au/SiO_2_ at 510 nm as a function of temperature, with linear fit to the data within 95% prediction band.

Figure 12a presents a detailed analysis of the optical responses of different characteristic peaks of LSPR under normal operation temperatures ranging from room temperature to 200 °C. The analysis shows both ascending and descending trends at different peaks for the Au/TiO_2_ sensor. Among these peaks, the 700 nm peak exhibits the most stable response over an extended range, whereas the 450 nm peak demonstrates better sensitivity at temperatures below 200 °C. Moreover, the multiple LSPR features presented by Au/TiO_2_ reflection spectra offer a unique potential of decoding simultaneous multivariate sensing beyond the temperature measurand alone, as shown in prior work [21,45]. Sensitivities of two sensing materials are recorded in real time in Figure 12b, where the Au/TiO_2_ sensor is interrogated at 700 nm and the Au/SiO_2_ sensor at 510 nm. The percentage changes in relative reflectance as a function of temperature are plotted to build a comparative case. At the respective interrogation wavelengths, the Au/SiO_2_ sensor yielded a higher averaged temperature sensitivity of 0.025%/°C than that of Au/TiO_2_ at around 0.01%/°C, at respective wavelengths. The response linearity of the two sensors can be represented by the $R^{2}$ value of the linear fit, which is 0.97428 and 0.97367 for Au/TiO_2_ and Au/SiO_2_, respectively. The observed quantitative experimental results are impacted by experimental details of the film morphologies and microstructure, which is expected to explain deviations from the quasistatic modeling approximation for Au/TiO_2_.

**Figure 13:**
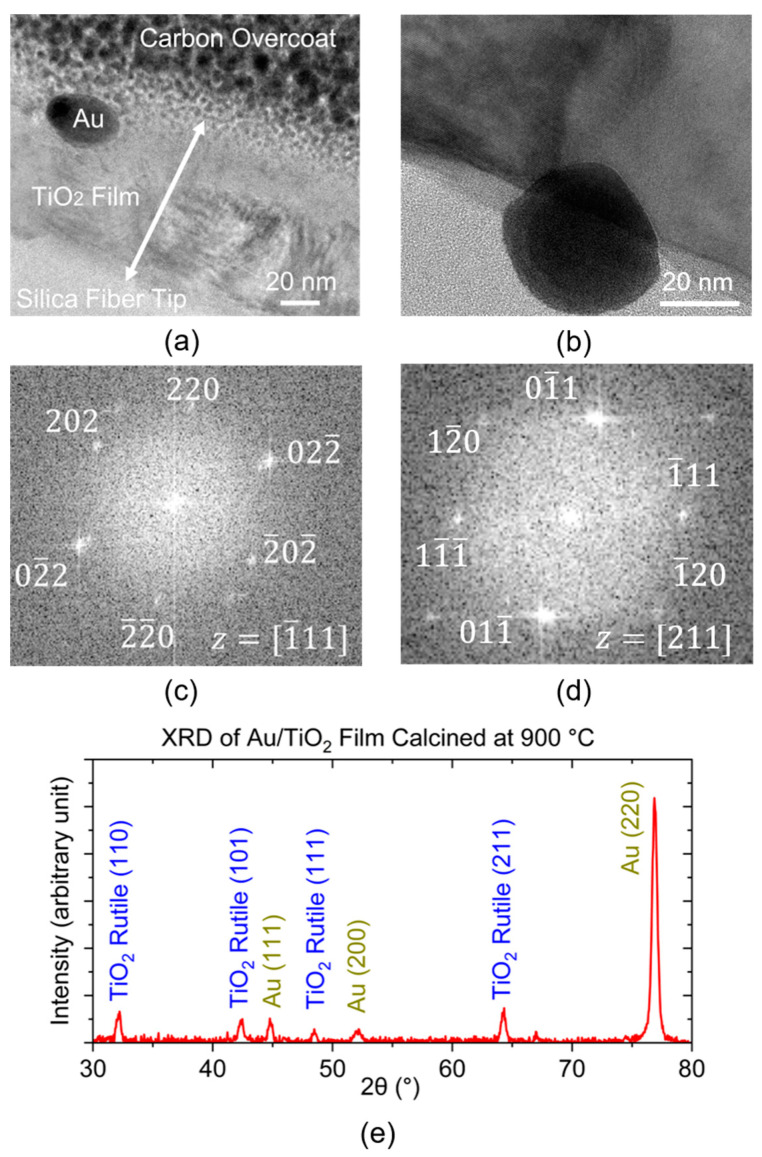
(**a**) Cross-sectional TEM image of post-temperature-sensing Au/TiO_2_ film, with Au NP embedded in the TiO_2_ matrix. (**b**) HRTEM image of a representative Au nanoparticle at the interface between the silica fiber tip and TiO_2_. (**c**) FFT pattern obtained from the Au nanoparticle in (**b**) along the zone axis $\left[\bar{1}11\right]$. (**d**) FFT pattern of TiO_2_ in the surrounding region of the Au nanoparticle along the zone axis [211]. (**e**) Grazing incidence XRD pattern of a reference Au/TiO_2_ planar sample on quartz disc made by the same recipe as the fiber sample in (**a**).

To better understand the film microstructure, post-experimental fiber samples of the Au/TiO_2_ sensors are studied with TEM (Hitachi High-Tech, Toronto, Ontario, Canada) images of a 5-μm piece of the film using FIB-based lift-out method. The actual film thickness on the fiber tip is verified by the cross-sectional TEM image in Figure 13a, showing that the Au/TiO_2_ film thickness is around 80 to 85 nm. The crystalline phases of the post-sensing film are confirmed with the high-resolution TEM (HRTEM) image of the Au nanoparticle and TiO_2_ presented in Figure 13b. Diffraction patterns of crystals can be seen after performing Fast-Fourier transform (FFT) on the HRTEM image. Patterns in Figure 13c, obtained along the $\left[\bar{1}11\right]$ zone axis, confirms the Face-Centered-Cubic (FCC) phase of the Au nanoparticle, whereas TiO_2_ is confirmed to be in a stable rutile phase, as shown in Figure 13d, along the [211] zone axis. In addition, this agrees with the X-ray diffraction (XRD) patterns conducted on nanocomposite thin film planar samples, shown in Figure 13e. XRD experiment is performed at grazing incidence with an Empyrean X-ray Diffractometer (Malvern Panalytical, Malvern, UK) using a cobalt source. A close comparison between the experimental XRD patterns and the standard XRD patterns of Au and TiO_2_ shows that the Au/TiO_2_ nanocomposite calcined at 900 °C is rutile phase dominated and showing the major Au FCC diffraction peaks.

## 5. Conclusions

We have experimentally confirmed the developed LSPR optical waveguide model for two exemplar Au-incorporated nanocomposite thin films fabricated in a simple reflection fiber probe configuration by an optical circulator. Physics-based optical waveguide modeling has proved to be an essential tool to benchmark the optical sensing performance for fiber optic sensors using functional material cladding. Both Au/TiO_2_ and Au/SiO_2_ nanocomposite sensors exhibit reasonable agreement, with Au/SiO_2_ being the more readily validated one in practice and therefore appears to have greater potential sensitivity for use as a reflection fiber probe for point temperature sensing in electrical power facilities. Au/TiO_2_, on the other hand, shows potential in multi-parameter sensing by identifying different representative LSPR absorption bands in the reflection spectrum. This can be particularly important for challenging environments where both temperature-rise and off-gassing are convoluted events that indicate imminent failures. With the experimental demonstrations up to 500 °C and potential for even higher temperatures, both sensing materials also show capability to be deployed in extreme temperatures and harsh environments for relevant applications. From a practical deployment perspective, the concept of low-cost interrogators based on a simple dual-stage TIA circuit with customized gain and filtering for intensity-based fiber temperature sensors has been demonstrated, with an additional advantage of wireless signal transmission to prevent hazardous high-voltage contacts during field equipment monitoring. The reflection configuration of the sensor probe itself also provides a geometrical advantage for the ease of installation. Future exploration in this topic can be expanded to further optimize both the temperature sensitivity and accuracy by incorporating bimetallic nanoparticles in stable oxide matrices. The temperature-dependent electron and phonon scattering based on plasmon coupling of different noble metals can provide more control parameters for the tunability of functional thin-film-based fiber optic temperature sensors. In addition, bivariate temperature and gas sensing can be studied using metal NP-incorporated thermo-optic dielectrics with high selectivity towards targeted gas species.

## Figures and Tables

**Table 1:** Model parameters used to generate optical constants and dielectric functions of Au nano particles in Figure 8.

Model Parameter of Au NP	Value
High frequency dielectric constant ($\varepsilon_{\infty }$)	1.5
Plasma frequency at room temp. ($\omega_{p}^{o}$)	$1.418\times 10^{16}$ rads
Thermal expansion coefficient ($\gamma_{e}$)	$1.42\times 10^{-5}{\;K}^{-1}$
Fermi velocity of free electrons ($v_{F}$)	1.4×106 ms
Assumed averaged Au NP size ($r_{np}$)	20 nm
Scattering probability (Γ)	0.55
Umklapp fractional scattering (∆)	0.77
Fermi energy ($E_{F}$)	5.53 eV
Debye temperature ($T_{D}$)	185 K
Frequency constant ($\omega^{0}$)	$1.0698\times 10^{14}$ rads

## Data Availability

The data presented in this study are available within the article.
